# Fast adaptation of tropical diatoms to increased warming with trade-offs

**DOI:** 10.1038/s41598-018-36091-y

**Published:** 2018-12-11

**Authors:** Peng Jin, Susana Agustí

**Affiliations:** 1 King Abdullah University of Science and Technology (KAUST), Red Sea Research Center (RSRC), Thuwal, 23955-6900 Saudi Arabia; 2 School of Environmental Science and Engineering, Guangzhou University, Guangzhou, 510006 China

**Keywords:** Microbial biooceanography, Microbial biooceanography, Microbial biooceanography, Microbial ecology, Microbial ecology

## Abstract

Ocean warming with climate change is forcing marine organisms to shift their distributions polewards and phenology. In warm tropical seas, evolutionary adaptation by local species to warming will be crucial to avoid predicted desertification and reduction in diversity. However, little is known about the adaptation of phytoplankton in warm seas. Across the ocean, diatomic microalgae are the main primary producers in cold waters; they also contribute to tropical communities where they play a necessary role in the biological pump. Here we show that four species of diatoms isolated from the tropical Red Sea adapted to warming conditions (30 °C) after 200–600 generations by using various thermal strategies. Two of the warming adapted species increased their optimal growth temperature (*T*_*opt*_) and maximum growth rate. The other two diatoms did not increase *T*_*opt*_ and growth, but shifted from specialist to generalist increasing their maximum critical thermal limit. Our data show that tropical diatoms can adapt to warming, although trade offs on photosynthetic efficiency, high irradiance stress, and lower growth rate could alter their competitive fitness. Our findings suggest that adaptive responses to warming among phytoplankton could help to arrest the sharp decline in diversity resulting from climate change that is predicted for tropical waters.

## Introduction

Ocean warming with climate change is occurring at an unprecedented rate as a result of increasing loads of atmospheric CO_2_ and other greenhouse gases^1^. Earth system models predict that ocean warming will result in a reduction in marine primary productivity (up to 20%) throughout the twenty-first century and driven by rising temperatures that exceed the limits of thermal tolerance^2,3^ and nutrient limitation in a warmer, more stratified ocean^4^. Oceanic primary production is accomplished by photosynthetic microorganisms^5^. Their short generation times and high population densities make evolutionary responses to climate change possible^6^. There is growing evidence of adaptation by phytoplankton through evolutionary responses to global change drivers, such as elevated CO_2_^7–10^, pollutants^11^, and temperature^12,13^. For example, previous studies reported fast adaptation of phytoplankton to warming after 80–450 generations^10,12^. Latitudinal patterns in thermal growth responses suggest that phytoplankton are adapted to environmental temperature, with tropical species showing optimum growth at the high *in situ* oceanic temperatures^14^. Rising temperatures leading to poleward shifts in the thermal niches of phytoplankton has been predicted by the end of this century along with a sharp decline in tropical phytoplankton diversity in the absence of evolutionary responses to warming^14^. Thus evolutionary responses of phytoplankton to warming will be essential to avoid a reduction in the biodiversity of the tropical ocean where replacement by temperate species is not expected. Diatomic algae are a relevant group contributing largely to oceans primary production and biogeochemical processes. Diatoms dominate primary producers in cold waters, and although are not dominant in tropical waters still play there a major role in the biological pump and other biogeochemical processes^15,16^. Diatoms have lower activation energies (i.e. less sensitive to increasing temperature) than other phytoplankton groups^17^, but its adaptation to warming will be crucial to maintain an efficient biological pump in the tropical ocean^16^.

Thermal and ecological theory describe that organisms’ adaptation to temperature could be accomplished by different strategies resulting in different performances. Organisms may adapt by showing “horizontal shifts” in thermal optimum and thermal limits, and therefore warm-adapted organisms can achieve the same maximum performance of growth as cold-adapted ones^18,19^. The “Hotter is better” hypothesis assumes that organisms’ performance will increase when warm adapted because temperature activates growth and other metabolic processes, and organisms adapted to lower temperatures are predicted to have lower maximum growth performances^18,19^. Then, the “Hotter is better” hypothesis predicts that organisms will increase growth together with the increase in the thermal optimum^20,21^. Organisms adapting to temperature can however move from “specialist” (i.e. growth is maximum at the optimum temperature) to “generalist” (maximum growth could be realized in a range of temperatures) and vice versa, with associated trade-offs in growth or other metabolic processes. If such trade-offs exist, and there is selection for an increased temperature range, specialist-generalist trade-offs would result in lower maximum performance for growth in warm-adapted organisms^18^. Thermal adaptation could generate a higher and broader growth curve (i.e. reaction norm), increasing the temperature range^19^. This adaptation is referred to as “hotter is broader and better” and will represent the win-win adaptation when organisms increased maximum growth and the breadth of reaction norm without trade-offs. For warm-adapted organism, as those from the tropical seas, it is unclear what type of thermal adaptations could experience when increasing warming will force thermal selection constraining performance.

In order to test adaptation to warming temperatures of warm-adapted tropical organisms, we conducted a long-term experiment on four diatomic phytoplankton species, *Chaetoceros tenuissimus, Chaetoceros* sp., *Thalassiosira* sp. and *Synedra* sp., isolated from the warm surface waters of the Red Sea. The four species of diatoms were maintained in the laboratory for about six months (~200–600 generations) at 26 °C (ambient temperature control) and at 30 °C (experimental warming conditions). 26 °C represents the mean Red Sea surface temperature (SST) for the 1982–2015 period (Fig. S1). For the projected warming conditions at 30 °C (mean Red Sea temperature +4 °C), we followed the high-emission scenario (RCP 8.5) projected for the turn of the next century (2100) by IPCC in 2014, that will represent a global mean warming ranging between 2.6 and 4.8 °C. The choice of ambient control temperature is problematic when considering interannual and seasonal variability^22^ as the sea surface temperature (SST) of Red Sea range from 21 to 32 °C^23^. However, phytoplankton species can adapt their realized niches to track average increases in water temperature regardless of annual and seasonal fluctuations, so the mean SST should be a reliable experimental control value^24^.

We investigated the thermal adaptation strategies of the four diatoms by combining experimental evolution with measurements of fundamental physiology (Fig. S2). The four species showed a fast adaptation to warmer temperature after 200–600 generations. Two of the species increased their optimal growth temperature (*T*_*opt*_) and maximum growth rate. The other two diatoms did not increase *T*_*opt*_ and growth, but shifted from specialist to generalist increasing their maximum critical thermal limit. However, none of the species was able to improve both and did not experience a “win-win” adaptation. The results suggested that despite adaptation was fast the trade-offs associated constrained growth or photophysiological performance.

## Results

After the six-month temperature selection period, *Chaetoceros* sp. and *Thalassiosira* sp. produced a similar number of generations of 439 and 440 generations and 413 and 393 generations for the experimental ambient and warming conditions, respectively. Due to its higher specific growth rate, *C. tenuissimus* produced nearly 600 generations (ambient: 556 generations, warming: 570 generations), whereas *Synedra* sp. produced only 200 generations (ambient: 188 generations, warming: 194 generations) due to its lower growth rate.

**Figure 1:**
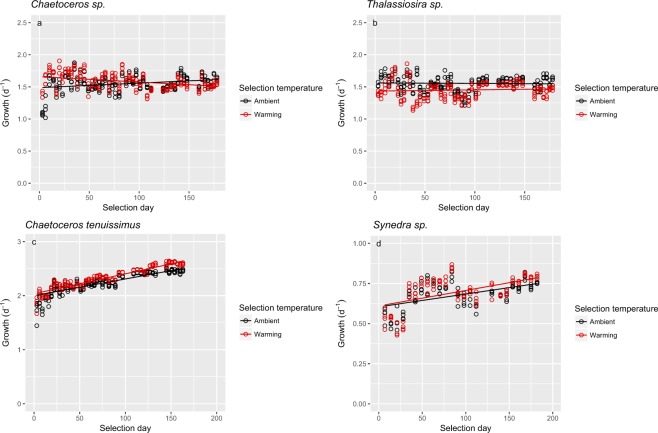
Growth rate trajectories for *Chaetoceros* sp. (**a**), *Thalassiosira* sp. (**b**), *Chaetoceros tenuissimus* (**c**) and *Synedra* sp. (**d**) at ambient (black symbols) and warming (red symbols) selection temperatures. Solid lines show growth trends based on the fixed effects of a linear mixed effect model (see Methods).

We used the mixed-effects model (Table 1) to understand the selection effect of time and temperature on the four species. Increasing temperature significantly affected the growth of all four species of diatoms. Selection time (*F* = 16.45, *p* < 0.001) and selection temperature (*F* = 42.61, *p* < 0.001) as well as the interaction of selection time and selection temperature (*F* = 189.34, *p* < 0.001) significantly affected the growth of *Chaetoceros* sp. (Fig. 1a, Table S1). Increased temperature increased the growth of *Chaetoceros* sp. at the beginning of the selection period, while this positive effect was reversed toward the end of selection period (Fig. 1a). Among *Thalassiosira* sp., the growth of warming-adapted cells was significantly lower than that of ambient-adapted cells (*F* = 142.56, *p* < 0.001) (Fig. 1b, Table S1). Among *C. tenuissimus*, increasing temperature increased the growth (*F* = 543.58, *p* < 0.001), and this positive effect was amplified by selection time (*F* = 39.00, *p* < 0.001) (Fig. 1c). Similarly, the growth of *Synedra* sp. was significantly affected by selection temperature (*F* = 29.85, *p* < 0.001) (Fig. 1d), with a positive effect on growth throughout the selection period (*F* = 2.92, *p* = 0.0889) (Fig. 1d). While two of the phytoplankton species (*C. tenuissimus* and *Synedra* sp.) benefitted from the temperature increase, the other two (*Chaetoceros* sp. and *Thalassiosira* sp.) did not.

**Table 1:** Linear mixed effects analysis for trajectories of the specific growth rate.

Species	Model structure	df	AIC	Log Lik	*L*-ratio	*p*
*Chaetoceros* sp.	Random effect structure					
	Random = ~1|id					
	Correlation structure					
	corARMA(q = 1), varPower(~temperature)					
	Fixed effect structure					
	**Growth~1** + selection day*Tem	8	−589	303		
	Growth~1 + selection day + Tem	7	−485	250	106	<0.0001
	Growth~1 + selection day	6	−472	242	15	0.0001
*Thalassiosira* sp.	Random effect structure					
	Random = ~1|id					
	Correlation structure					
	Corr.structure = corAR1(),					
	varPower()					
	Fixed effect structure					
	**Growth~1** + selection day*Tem	8	−564	290		
	Growth~1 + selection day + Tem	7	−560	287	5.9	0.0149
	Growth~1 + selection day	6	−477	245	85	<0.0001
*Chaetoceros tenuissimus*	Random effect structure					
	Random = ~1|id					
	Correlation structure					
	Corr.structure = corAR1(),					
	varPower()					
	Fixed effect structure					
	Growth~1 + selection day*Tem	8	−956	486		
	Growth~1 + selection day + Tem	7	−929	471	30	<0.0001
	Growth~1 + selection day	6	−725	368	206	<0.0001
*Synedra* sp.	Random effect structure					
	Random = ~1|id					
	Correlation structure					
	Corr.structure = corAR1(),					
	varExp()					
	Fixed effect structure					
	**Growth~1** + selection day*Tem	8	−556	286		
	Growth~1 + selection day + Tem	7	−550	282	8.2	0.0042
	Growth~1 + selection day	6	−532	272	20	<0.0001

Random effects on the slope and intercept were determined at the level of replicates nested within the selection temperatures. Analyses show that growth rates changed significantly over time and that growth trajectories were significantly different between selection temperatures. The most parsimonious model is highlighted in bold.

**Figure 2:**
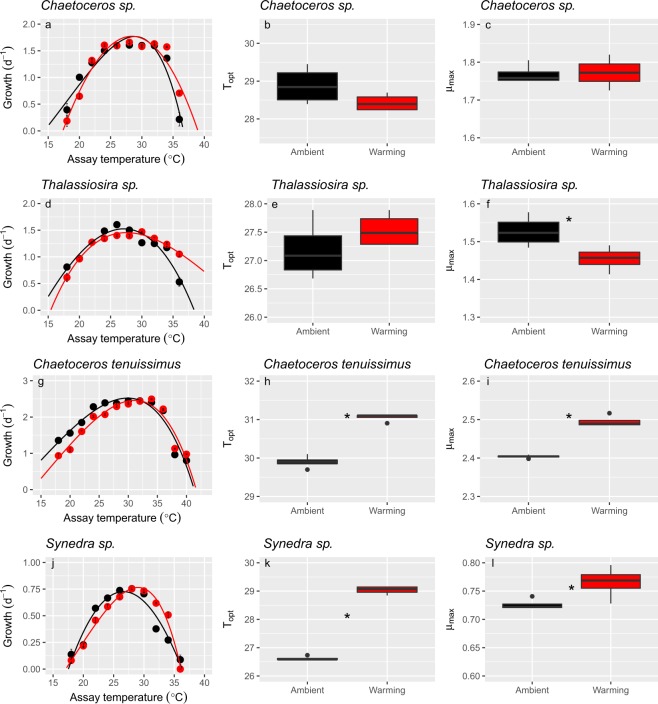
Patterns of thermal adaptation in *Chaetoceros* sp. (**a**–**c**), *Thalassiosira* sp. (**d**–**f**), *Chaetoceros tenuissimus* (**g**–**i**) and *Synedra* sp. (**j**–**l**) at ambient (black symbol) and warming (red symbol) selection temperatures. a, d, g and j, Thermal reaction norms for growth rates across a wide range of temperatures. Solid lines show the thermal reaction norms based on the thermal model. b, e, h and k, Box and whisker plots of the replicate-level (n = 4) optimal temperature for growth (*T*_opt_) at ambient and warming selection temperatures. c, f, i and l, Box and whisker plots of the replicate-level (n = 4) maximum growth rate (μ_max_) at ambient and warming selection temperatures. Asterisks indicate significant differences between ambient and warming treatments based on the Students’ t-test.

Adaption to increased temperature was evident in changes in the growth rates in relation to thermal performance. In *Chaetoceros* sp., the maximum growth rate of warming-adapted cells and *T*_opt_ were similar to that of the ambient-adapted strain (Fig. 2a). However, the critical thermal minimum (*CT*_*min*_) and critical thermal maximum (*CT*_*max*_) shifted significantly in warming-adapted cells (Table 2, Table S2). Similarly, warming-adapted *Thalassiosira* sp. experienced relatively higher growth rates at high temperatures (Fig. 2d). In parallel, the *CT*_*max*_ shifted in warming-adapted *Thalassiosira* sp. cells (ambient: 38.44 ± 0.6 °C; warming: 47.34 ± 2.32 °C) and the thermal breadth also increased (expressed as *B*_80_) (Table 2, Fig. 2 and Table S2). The μ_max_ of warming-adapted *Thalassiosira* sp. cells significantly decreased when compared with that of ambient cells (*t*-test, *t* = 2.763, *p* = 0.033) (Table S2, Fig. 2f), indicating the cost of thermal adaptation. There were no significant changes in *T*_opt_ between ambient and warming-adapted *Thalassiosira* sp. cells (Fig. 2e). However, there were significant shifts in *T*_opt_ in *C. tenuissimus* (ambient: 29.9 ± 0.16 °C; warming: 31.1 ± 0.10 °C, Fig. 2) and *Synedra* sp. (ambient: 26.6 ± 0.08 °C; warming: 29.0 ± 0.14 °C, Fig. 2), suggesting that a different thermal adaptation occurred in these two species (Stats see in Table S2) (Fig. 2h,k). Furthermore, thermal breadth narrowed in warming-adapted *C. tenuissimus* cells, mostly due to the increase in *CT*_*min*_ (Table 2). The μ_max_ of *C. tenuissimus* and *Synedra* sp. both increased significantly in warming-adapted cells (Fig. 2i,l, and Table S2).

**Table 2:** The critical thermal minimum (*CT*_*min*_, °C), critical thermal maximum (*CT*_*max*_, °C) and 80% performance breadth (*B*_80_, °C) of *Chaetoceros* sp., *Thalassiosira* sp., *Chaetoceros tenuissimus* and *Synedra* sp. under ambient and warming conditions.

Species	Treatment	*CT*_*min*_	*CT*_*max*_	*B*_80_
*Chaetoceros sp*.	Ambient	13.66 ± 2.41^a^	36.76 ± 0.56^a^	8.93 ± 0.70^a^
	Warming	17.27 ± 0.83^b^	39.02 ± 0.19^b^	9.72 ± 0.26^a^
*Thalassiosira sp*.	Ambient	13.47 ± 1.53^a^	38.44 ± 0.60^a^	10.90 ± 0.70^a^
	Warming	15.28 ± 0.93^a^	47.34 ± 2.32^b^	13.22 ± 1.00^b^
*Chaetoceros tenuissimus*	Ambient	10.40 ± 0.28^a^	41.31 ± 0.10^a^	12.66 ± 0.16^a^
	Warming	13.37 ± 0.93^b^	41.66 ± 0.12^b^	11.71 ± 0.19^b^
*Synedra sp*.	Ambient	17.50 ± 0.58^a^	36.31 ± 0.56^a^	8.40 ± 0.45^a^
	Warming	17.05 ± 0.66^a^	36.12 ± 0.08^a^	7.80 ± 0.23^a^

These three thermal reaction traits were derived from the nonlinear curve fitting according to equation (4) based on their thermal reaction norms (Fig. 2). Data are presented as means ± SE. The superscripts represent the significant difference between ambient and warming treatments in each species based on a Student’s *t*-test.

**Figure 3:**
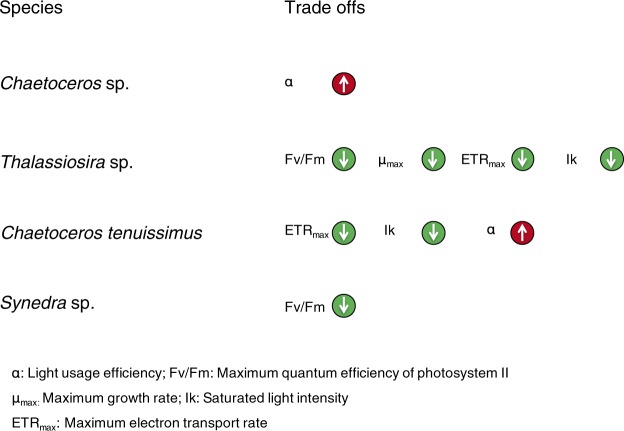
Summary of the trade-offs that are associated with thermal adaptations in *Chaetoceros* sp., *Thalassiosira* sp., *Chaetoceros tenuissimus* and *Synedra* sp. α: Light usage efficiency; Fv/Fm: Maximum quantum efficiency of photosystem II; μ_max:_ Maximum growth rate; Ik: Saturated light intensity; ETR_max_: Maximum electron transport rate.

Any cost of thermal adaptation could be also reflected in differences in photosynthetic parameters. The maximum quantum efficiency (Fv/Fm) of photosystem II (PSII) decreased significantly in warming-adapted *Thalassiosira* sp. and *Synedra* sp. when compared with that of ambient-adapted cells assayed in their ancestral environment (Fig. S3b,d). The maximum electron transport rate (ETR_max_) of warming-adapted *Thalassiosira* sp. cells were significantly lower than the ambient-adapted cells when both assayed at warming temperature (Fig. S4b). For warming adapted *C. tenuissimus*, ETR_max_ significantly decreased compared with that of the ambient-adapted cells assayed at the ambient temperature (Fig. S4c). Light usage efficiency, α, showed a significant increase in the warming-adapted *Chaetoceros* sp. cells assayed at the warming temperature compared with that of ambient-adapted cells assayed at the ambient temperature (Fig. S5a). Moreover, the warming-adapted cells of *C. tenuissimus* exhibited significantly higher α than did ambient-adapted cells when both were subjected to warming assay conditions (Fig. S5c), suggesting that the warming-adapted cells could maintain sufficiently high photosynthetic capacity at warm temperatures to support higher growth rates. Costs due to thermal adaptation were also reflected in the measure of saturated light intensity (Ik), with the warming-adapted *C. tenuissimus* and *Thalassiosira* sp. exhibiting significantly lower Ik when assayed under warming conditions compared with ambient-adapted cells (Fig. S6c), suggesting a decrease in tolerance to high irradiance among warming-adapted cells. The summary of the photophysiological responses pointed to *Thalassiosira* sp. as the species that suffered the larger trade–offs after adaptation to warming (Fig. 3).

## Discussion

**Figure 4:**
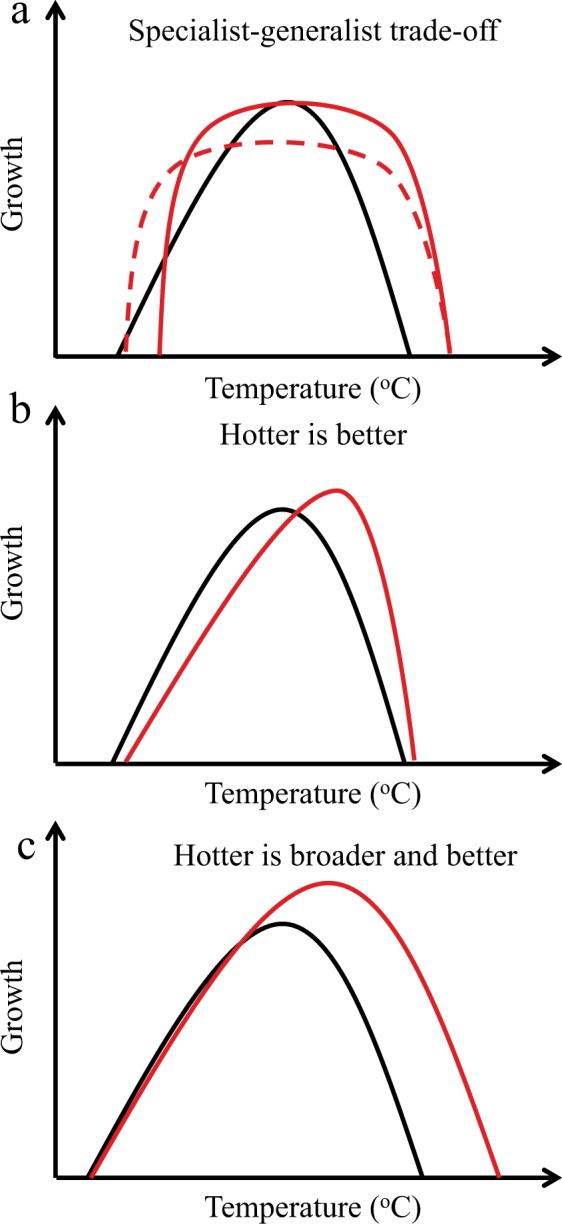
Thermal adaptation optimality models for tropical diatoms. (**a**) Change from specialist to generalist trade-off. *Chaetoceros* sp. and *Thalassiosira* sp. conformed to this adaptation model in which the optimal temperature for growth did not shift, but the thermal niche width in warming-adapted cells (red lines) was wider than that of ambient-adapted cells (black line). The adaptation trade offs of *Thalassiosira* sp. (dashed red line) imply decreasing maximum growth rates. (**b**) The “hotter is better” model predicts that populations adapted to higher temperatures (red line) increased their optimal temperature for growth and their maximum growth rate with respect to ancestors (black line), as observed in *C. tenuissimus* and *Synedra* sp. (**c**) The “hotter is better and broader” model was not utilized by diatoms in the present study. This model implies no trade-offs for adaptation to higher temperatures, resulting in increased maximum growth rate and optimal temperature and a wider thermal niche.

Overall, our study provides evidence that phytoplankton from the tropical Red Sea demonstrated the capacity to adapt fast to ongoing warming. However, the tested diatoms exhibited differing thermal adaptation capacities. The adaptation strategies of *Chaetoceros* sp. and *Thalassiosira* sp. followed a pattern of changing from “specialist” to “generalist” (Fig. 4a) by shifting the critical thermal minimum (*CT*_*min*_) and maximum (*CT*_*max*_) in warming-adapted cells, with no shifts in the optimal temperature. In this trade-off model, the phytoplankton improved their growth rates at higher temperatures and functioned over a wider range of high temperatures, whereas they reduced their growth capacity at the lowest temperatures. Furthermore, costs are likely to be associated with shifts of critical thermal limits^25^ because adaptations of enzymes and membranes to high temperatures may result in maladaptations to low temperatures^26,27^. The “hotter is better” hypothesis assumes that low temperature slows rates of biochemical reactions and organisms adapted to lower temperatures are predicted to have lower maximum performances (e.g., sprinting speeds, fitness, and growth rates) than those adapted to higher temperatures^18^. Then organisms adapted to warming therefore should have higher maximum growth rates and optimal growth temperatures than those adapted to low temperatures^18,19^. *C. tenuissimus* and *Synedra* sp. utilized a “hotter is better” strategy^25,27^ (Fig. 4) to adapt to warming by shifting their *T*_opt_ and increasing their μ_max_ under warming conditions. However, *C. tenuissimus* showed trade-offs in their photosynthetic performance increasing α and decreasing the light intensity to saturate photosynthesis, reducing tolerance to high light. Successful long time (one decade) adaptation to warming of the microalgae *Chlamydomonas reinardtii*, resulted in a significant increase in *T*_*opt*_ and maximum growth rate, accompanied by an increase in the photosynthetic capacity^13^. In fact, *C. reinardtii* showed the “hotter is better and broader” model, which implied no trade-offs^19^. Furthermore, *C. tenuissimus* and *Synedra* sp. benefitted from the temperature increase by increasing their growth, while the other two species of *Chaetoceros* sp. and *Thalassiosira* sp. did not. Warming adapted *Thalassiosira* sp. also showed a reduction on the photosynthetic performance at high light. Although the underlying mechanisms for the interspecific differences are unclear based on the present study, the various thermal adaptation strategies implied different trade offs. Furthermore, costs are likely to associate with shifts of critical thermal limits^26^.

Diatoms become the predominant phytoplankton in the Red Sea when nutrient availability increases^28,29^. Diatoms proportion increase in the North during winter, in the cyclonic eddies and largely in the South, the warmest area in the Red Sea where temperature ranges between 26 and 32 °C and nutrient availability increases through the influx of Gulf of Aden Intermediate Water^28–30^. Their adaptation capacities to warming, on one hand, may help them to maintain their presence and distribution in the tropical ocean, maintaining the biogeochemical processes sustained by diatoms^16,31,32^. On the other hand, the trade offs associated with the thermal adaptations may decrease their competitive fitness, and could reshape the communities.

A recent comparative analysis coupled with an eco-evolutionary species distribution model demonstrated that the geographic variation in thermal niches of phytoplankton closely matched the temperature regime of their natal environment^14^. This study suggests that rising temperatures will cause poleward shifts in the thermal niches of phytoplankton and a sharp decline in trophic phytoplankton diversity in the absence of evolutionary adaptation. Our long-term experiments on diatoms from a tropical sea suggested that ~200–600 generations (6 months) were sufficient to evoke adaptation to ocean warming. Our observations that the Red Sea diatoms could shift their optimal growth temperatures or critical thermal limits provide an empirical basis for parameterizing thermal evolution in eco-evolutionary models of phytoplankton dynamics. Our study suggests that rapid thermal adaptation may help to reduce the sharp decline in tropical phytoplankton diversity as projected in the absence of evolutionary responses.

Several recent studies have shown that adaptation to warming becomes evident after 80–100 generations^12,13^. However, experiments investigating evolutionary responses to environmental change in phytoplankton have thus far focused on phytoplankton from natal environments in the temperate and sub-tropical oceans. Our study builds on this knowledge by investigating adaptive responses in organisms isolated from one of the warmest seas in the world^23^. Our findings demonstrate that photosynthetic microorganisms living in oceanic extreme temperatures can adapt to future warming conditions. The tropical diatoms tested showed contrasting adaptation capacities with different strengths and weaknesses. Specialization to increased optimal temperatures optimizes responses against competitors at higher water temperatures, but the thermal niche allows a generalist species to persist in a changing thermal environment. However, these adaptation strategies involve costs for some species, associated with trade-offs, including reduction in the maximum growth or the photosynthetic capacity, that should be considered for projections (Fig. S7). Since the oceans are now undergoing multiple environmental changes including ocean acidification, deoxygenation, increased light exposure and decreased nutrient availability within the upper mixing layers^33^, the adaptation associated with such trade-offs may alter the resistance of a phytoplankton species to other stressors. At the same time, the thermal adaptation of phytoplankton under natural conditions might also be amplified or retarded by other environmental changes and it might also depend on co-evolutionary interactions with other species that remain difficult to predict.

Fast organisms’ adaptation will be required in fast changing environments. The Red Sea is warming at 0.17 ± 0.07 °C decade^−1^, exceeding the global rate^23^. Our results confirmed fast adaptation capacity to warming (i.e. within months) for Red Sea diatoms. Irwin *et al*.^24^ found that phytoplankton species in the CARIACO station in a 15-years time series adapted to a change of 1 °C over a decade^24^. Our study was conducted at fixed ambient and warming scenarios ignoring the temperature fluctuations in the natural environment and its influence on the rate of adaptation. Future studies with fluctuating treatment mimicking temperature variation in the region would contribute to help identify more accurate adaptation rates as well as examine thermal adaptation strategies and trade-offs.

In conclusion, our results suggest organisms from the warmest sea on earth can adapt fast to ongoing warming, on top of already experiencing thermal extremes, with trade-offs. Experiments examining evolutionary responses to increasing temperature in the ocean, like those performed in this study, provide important insights into responses of organisms to climate change. They also help to identify potential evolutionary winners and losers in the context of climate change. The thermal adaptation strategies reported in the present study and associated constrains to performance, may improve our knowledge on the ecological evolutionary responses to warming.

## Methods

### Species isolation and culture conditions

Four diatom species, *Chaetoceros* sp., *Thalassiosira* sp., *Chaetoceros tenuissimus* and *Synedra* sp., were isolated from coastal Red Sea waters near the Al Fahal Reef (22.2528°N, 38.9612°E). Surface water samples were collected and then were passed through a 45-μm filter. Approximately ten clonal cultures were established by single-cell isolation under a microscope. All cultures were maintained as batch cultures in filtered seawater that was taken from the same location and enriched with f/4 medium and silicate. The cultures incubated at 24 °C in a precise temperature-controlled incubator (Percival, United States). The cultures grew with a light: dark cycle of 12 h: 12 h under 50 μmol photons m^−2^ s^−1^.

After pure cultures of each species were established, mono-specific cultures of *Chaetoceros* sp., *Thalassiosira* sp., *Chaetoceros tenuissimus* and *Synedra* sp. were grown in 200 mL Erlenmeyer flasks at 26 ± 0.1 (experimental ambient, termed as ambient hereafter) and 30 ± 0.1 °C (experimental warming, termed as warming hereafter). The design of the warming temperature that ignoring the regional temperature variation (both annual and seasonal) in the Red Sea rather serves as a temperature increase by average as projected by high-emission scenario (RCP 8.5, IPCC 2014) for the turn of next century, to explore the extent the adaptive capacity. The cultures grew under 400 μmol photons m^−2^ s^−1^ with a light: dark cycle of 12 h: 12 h. Four independent replicated cultures (n = 4) were run semi-continuously for about 6 months under ambient and warming conditions by renewing the medium every 3 days for *Chaetoceros* sp., *Thalassiosira* sp. and *C. tenuissimus* and every 7 days for *Synedra* sp. due to their lower growth rate. This long-term nature of the experiments allowed the diatoms to adapt to the experimental temperature environment. The initial cell concentration was set at 1000 cells mL^−1^, and the medium was partially renewed every 3 or 7 days to restore the cell density to the initial level (i.e., growth batch cycle). Nutrients were not limiting as the cell abundances achieved at the end of the batch cycles were far from those expected at the stationary phase. Cell abundance was quantified every 3 days for *Chaetoceros* sp., *Thalassiosira* sp. and *C. tenuissimus* and every 7 days for *Synedra* sp., by examining the samples under an optical microscope (LEICA DMI 3000B-Germany) by hemocytometer. The specific growth rate, μ (d^−1^), during each batch growth cycle was calculated as

1 $${\rm{\mu }}=\frac{ln({C}_{1}-{C}_{0})}{({t}_{1}-{t}_{0})},$$

where *C*_1_ and *C*_0_ are the cell densities at times *t*_1_ and *t*_0_ (*t*_1_ − *t*_0_ = 3 or 7 days), respectively. The number of generations per transfer (g) is equivalent to the number of doubles and was calculated as follows:

2 $$g=\frac{{t}_{1}-{t}_{0}}{ln(2)/{\rm{\mu }}},$$

where *t*_1_ − *t*_0_ is the time interval of the transfer (d), ln (2)/μ is the doubling time (d) and μ is the specific growth rate (d^−1^).

### Thermal growth curves

The thermal growth responses for all four diatoms adapted to ambient and warming conditions for about 6 months were determined at ten (*Chaetoceros* sp., *Thalassiosira* sp. and *Synedra* sp.) or twelve (*C. tenuissimus*, depending on its high thermal capacity) assay temperatures. At the end of the long-term selection period, cultures of ambient and warming conditions were inoculated into 200 mL flasks at an initial cell density of 1000 cells mL^−1^, and then incubated at 18, 20, 22, 24, 26, 28, 30, 32, 34 and 36 °C (*Chaetoceros* sp., *Thalassiosira* sp. and *Synedra* sp.) and 18, 20, 22, 24, 26, 28, 30, 32, 34, 36, 38 and 40 °C for species *C. tenuissimus*. To accommodate the different growth rates, batch cycles lasted 3 days for *Chaetoceros* sp. (~6–8 generations), *Thalassiosira* sp. (~5–7 generations) and *C. tenuissimus* (~9–11 generations) and 7 days for *Synedra* sp. (~7–8 generations) of the different temperatures. At the end of each growth cycle, the cell densities were determined by counting cell abundances under an optical microscope, and specific growth rates (μ) for each assay temperature were calculated from cell abundances versus time.

### Photosynthetic responses to temperature

Photosynthetic responses were measured at the end of each growth cycle using Pulse-Amplitude-Modulation (PAM) fluorometry (Phyto-PAM, Walz, Germany) at the ambient and warming temperatures in a reciprocal assay in which warming-adapted and ambient-adapted populations were compared against the respective non-adapted (i.e., 30 °C and 26 °C, for warming and ambient, respectively) and vice versa (Fig. S2). All the measurements were made in the middle of the light phase.

The maximum quantum yield (Fv/Fm) of photosystem II (PSII) was measured on samples that were adapted to the dark for 15 min and then determined by a saturated pulse (5000 μmol photos m^−2^ s^−1^). For the rapid light curve (RLC) measurements, we determined the relative electron transport rate (rETR) at 12 different light levels (1, 16, 32, 64, 164, 264, 364, 564, 764, 1064, 1364 and 1664 μmol photons m^−2^ s^−1^), each lasting for 20 s. The rETR (an arbitrary unit) was calculated as

3 $$rETR={\rm{\Phi }}\mathrm{PSII}\times 0.5\times \mathrm{PAR},$$

where ΦPSII is the photochemical quantum yield of PSII in light, PAR is the actinic light intensity (μmol photons m^−2^ s^−1^), and the factor 0.5 accounts for approximately 50% of all the absorbed energy allocated to PSII. RLC was fitted with the model proposed in ref.^34^. The photosynthetic parameters maximum electron transport rate (ETR_max_), light usage efficiency (α) and saturated light intensity (Ik) were derived from the fitted curves (Supplementary Methods).

### Statistical analyses

We used the linear mixed-effects model to quantify trajectories of specific growth rates under experimental ambient and warming temperatures. For the analysis, μ was considered the dependent variable, time (in days) and treatment temperature were the fixed effects, while slopes and intercepts were treated as random effects at the level of replicates nested within the selection temperature^35^. The significance of the parameters was assessed using likelihood ratio tests, comparing models with common slopes and intercepts for each selection temperature (Table 1). Comparisons of models were done using Akaike’s information criterion (AIC) and likelihood ratio tests on models fitted with restricted maximum likelihood (REML) for comparison of random effects, and maximum likelihood (ML) for comparison of fixed effects^36^.

The thermal reaction norms of the four diatoms adapted to the two treatment temperatures were assessed by applying the equation described in refs^14,37^:

4 $$f(T)=a{e}^{bT}[1-{(\frac{T-z}{w/2})}^{2}],$$

where specific growth rate, *f*, depends on temperature, *T*, and is defined as a function of the parameters *z*, *w*, *a* and *b*. *w* is the temperature niche width (the range of temperatures over which the growth rate is positive), while the other three (*z*, *a*, *b*) possess no explicit biological meaning but interact to influence the rate of increase in growth with temperature, the maximum growth rate and the optimum temperature for growth. Specifically, *z* determines the location of the maximum of the quadratic portion of this function. *a* and *b* are the Epply curve coefficient and Epply curve exponent, respectively. We estimated the critical thermal minimum (*CT*_*min*_, the lowest temperature at which the growth of phytoplankton is zero), the critical thermal maximum (*CT*_*max*_, the highest temperature at which the growth of phytoplankton is zero)^38^, the maximum growth rate (μ_max_), the optimal temperature for growth (*T*_opt_), identified as the temperature at which the growth rate is maximal, and thermal breadth (expressed as *B*_80_, 80% performance of the maximum growth rate breadth) by numerically maximizing the equation after estimating the parameter values for each replicate. We note that the estimated μ_max_ in ambient- and warming-adapted *C. tenuissimus* showed followed opposite trends with that of measured μ_max_. The measured μ_max_ values are presented in Fig. 2i. For the other three species, μ_max_ is given in the values estimated by equation (4). Student’s t-test was used to test the differences in *CT*_*min*_, *CT*_*max*_, *T*_opt_, μ_max_ and *B*_80_ between two temperature-adapted populations.

## Electronic supplementary material

Supplementary Information
