# Supplementary material for: Fast adaptation of tropical diatoms to increased warming with trade-offs
Source: Sci Rep. 2018 Dec 11;8:17771. doi: 10.1038/s41598-018-36091-y (PMC6289974; doi:10.1038/s41598-018-36091-y)
Supplement: Supplementary file 1 — Supplementary Information [file 41598_2018_36091_MOESM1_ESM.pdf]

Fast adaptation of tropical diatoms to increased warming with trade-offs

Peng Jin[1,2*], Susana Agustí[1]

[1] King Abdullah University of Science and Technology (KAUST), Red Sea Research Center (RSRC), Thuwal, 23955-6900, Saudi Arabia

[2] School of Environmental Science and Engineering, Guangzhou University, Guangzhou, 510006, China

[*] **Corresponding author**: pengjin@gzhu.edu.cn

Supplementary Methods

Photosynthetic responses to temperature

The maximum quantum yield (Fv/Fm) of photosystem II (PSII) was measured on samples adapted to the dark for 15 min and then determined by a saturated pulse (5000 μmol photos m^{-2} s^{-1}). For the measurement of rapid light curve (RLC), the relative electron transport rate (rETR) was determined at 12 different light levels (1, 16, 32, 64, 164, 264, 364, 564, 764, 1064, 1364 and 1664 μmol photons m^{-2} s^{-1}), each lasting for 20 s. The rETR (an arbitrary unit) was calculated as:

$$rETR = \Phi\text{PSII} \times 0.5 \times \text{PAR}, \qquad (1)$$

where ΦPSII is the photochemical quantum yield of PSII in light, PAR is the actinic light intensity (μmol photons m^{-2} s^{-1}), and the factor 0.5 accounts for approximately 50% of all the absorbed energy allocated to PSII. RLC was fitted by following model (ref 1):

$$y = \frac{x}{ax^2+bx+c}\ , \qquad (2)$$

where y is the rETR, x is the photon flux density of actinic light (μmol photons m^{-2} s^{-1}), a, b and c are the adjustment parameters. The maximum electron transport rate (ETR_{max}) was calculated as

$$\text{ETR}_{max} = \frac{1}{b+2\sqrt{ac}}\ , \qquad (3)$$

the light usage efficiency α was calculated as

$$\alpha = \frac{1}{c}\,, \qquad (4)$$

and the saturated light intensity (Ik) was calculated as

$$\mathrm{I}_k = \frac{c}{b+2\sqrt{ac}}. \qquad (5)$$

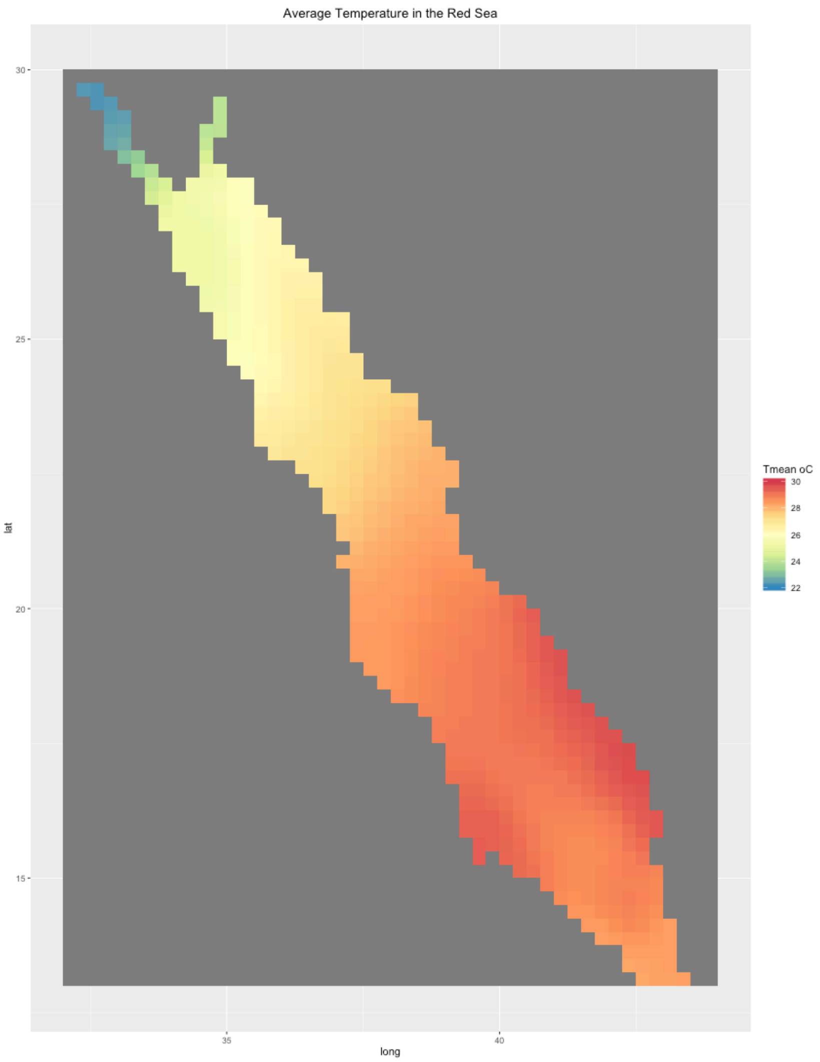

Fig. S1 Mean sea surface temperatures in the Red Sea from 1982-2015. The data was obtained from the National Aeronautics and Space Administration's National Climatic Data Center[2] at podaac.jpl.nasa.gov.

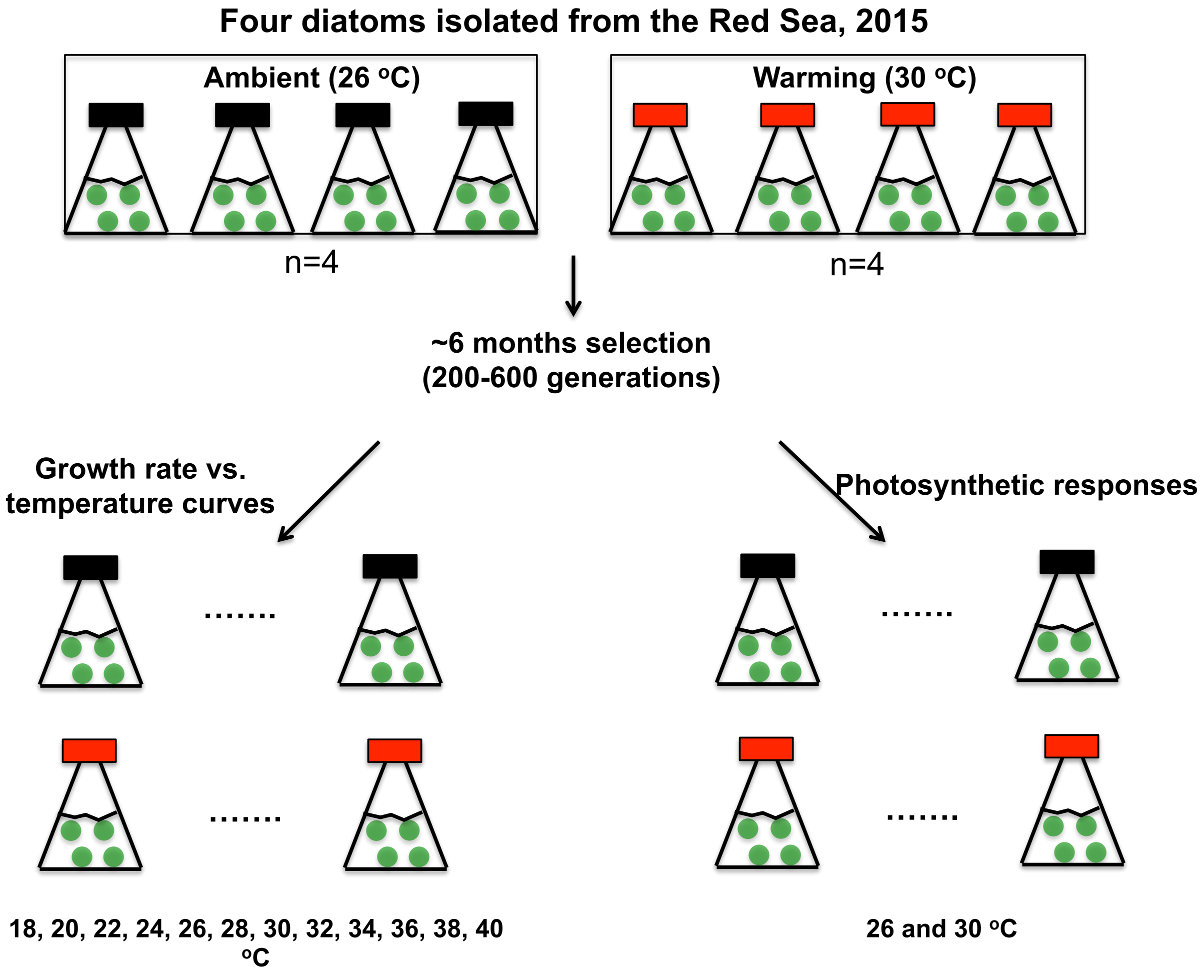

Fig. S2 General experimental setup of the selection and response experiments. Four replicates of the four diatom species were grown in two selection environments (ambient: 26 ºC; warming: 30 ºC) for about 6 months. At the end of the selection experiments, we analyzed the growth rates at a range of temperatures (from 18 to 40 ºC) and the photosynthetic performance (at ambient and warming temperatures).

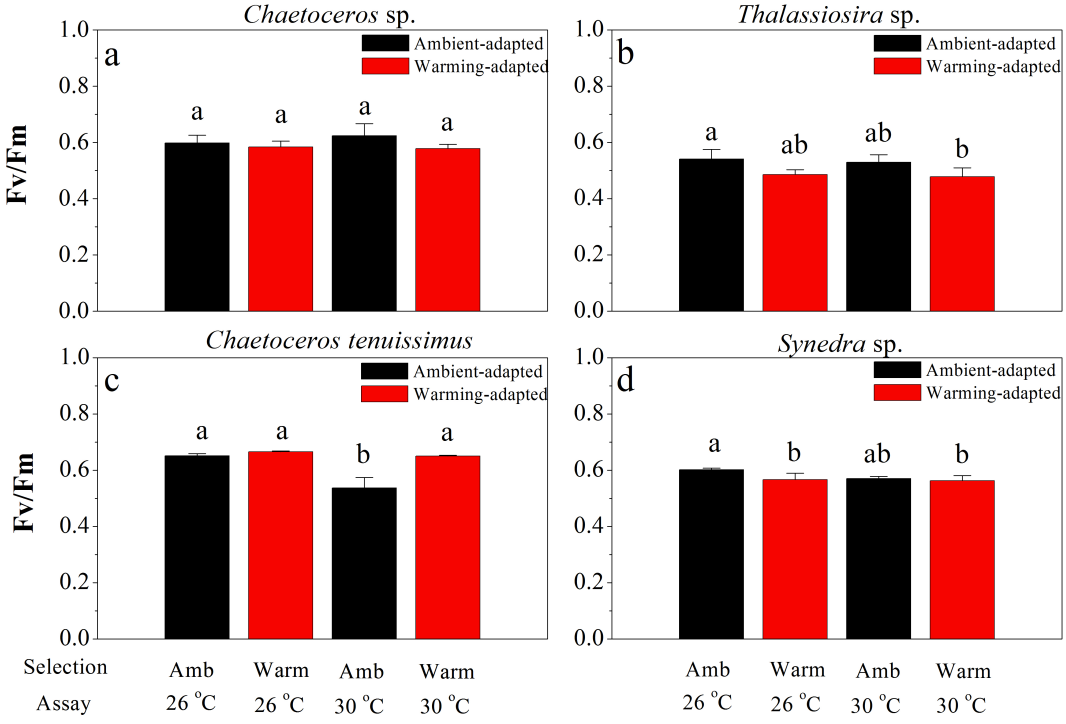

Fig. S3 Photosynthetic responses of maximum quantum efficiency (Fv/Fm) of photosystem II (PSII) of ambient- (26 °C) (black bars) and warming- (30 °C) (red bars) adapted *Chaetoceros* sp. (a), *Thalassiosira* sp. (b), *Chaetoceros tenuissimus* (c) and *Synedra* sp. (d) Cells were exposed to both ambient and warming temperatures. Data are mean ± SE of 4 measurements, and the different letters indicate significant differences between treatments.

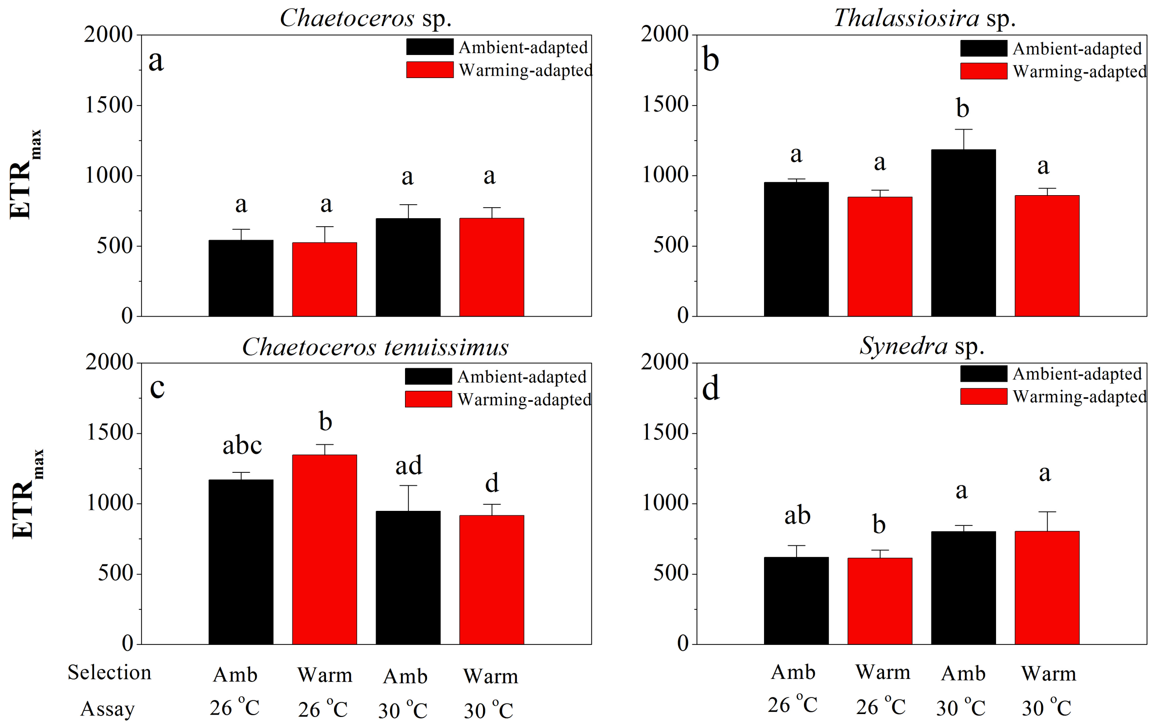

Fig. S4 Photosynthetic responses of maximum electron transport rate (ETR_{max}) of ambient- (26 °C) (black bars) and warming- (30 °C) (red bars) adapted *Chaetoceros sp* (a), *Thalassiosira sp* (b), *Chaetoceros tenuissimus* (c) and *Synedra* sp. (d) Cells were exposed to both ambient and warming temperatures. Data are mean ± SE of 4 measurements, and the different letters indicate significant differences between treatments.

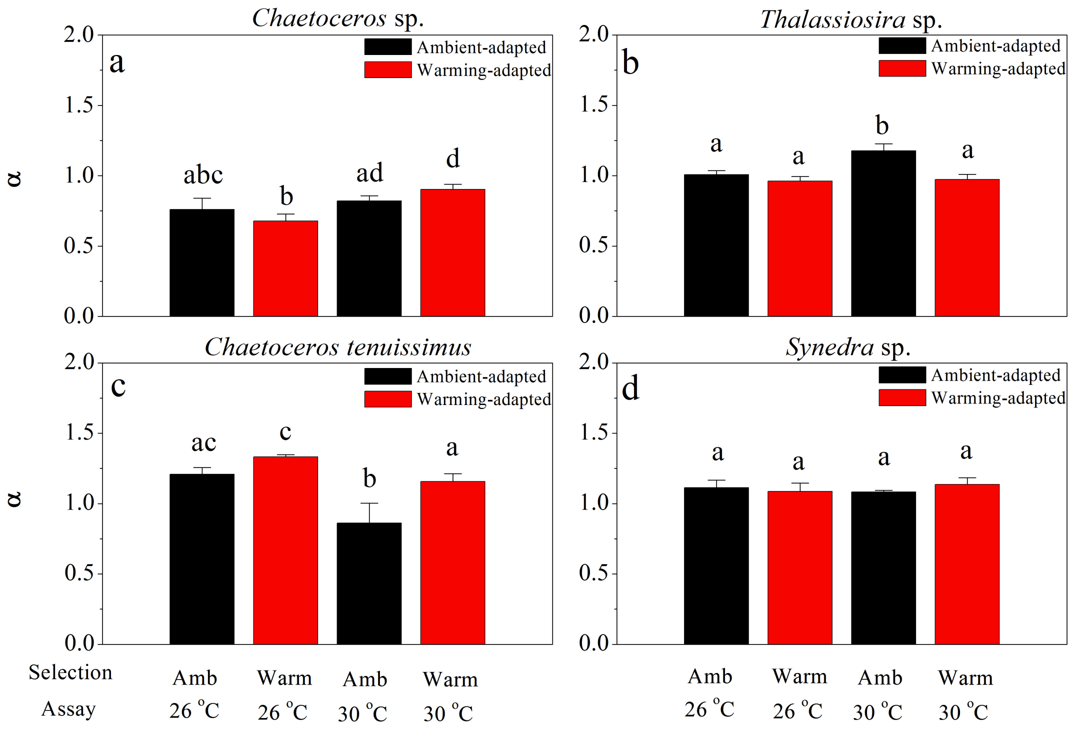

Fig. S5 Photosynthetic responses of light usage efficiency (α) of ambient- (26 °C) (black bars) and warming- (30 °C) (red bars) adapted *Chaetoceros sp* (a), *Thalassiosira sp* (b), *Chaetoceros tenuissimus* (c) and *Synedra* sp. (d) Cells were exposed to both ambient and warming temperatures. Data are mean ± SE of 4 measurements, and the different letters indicate significant differences between treatments.

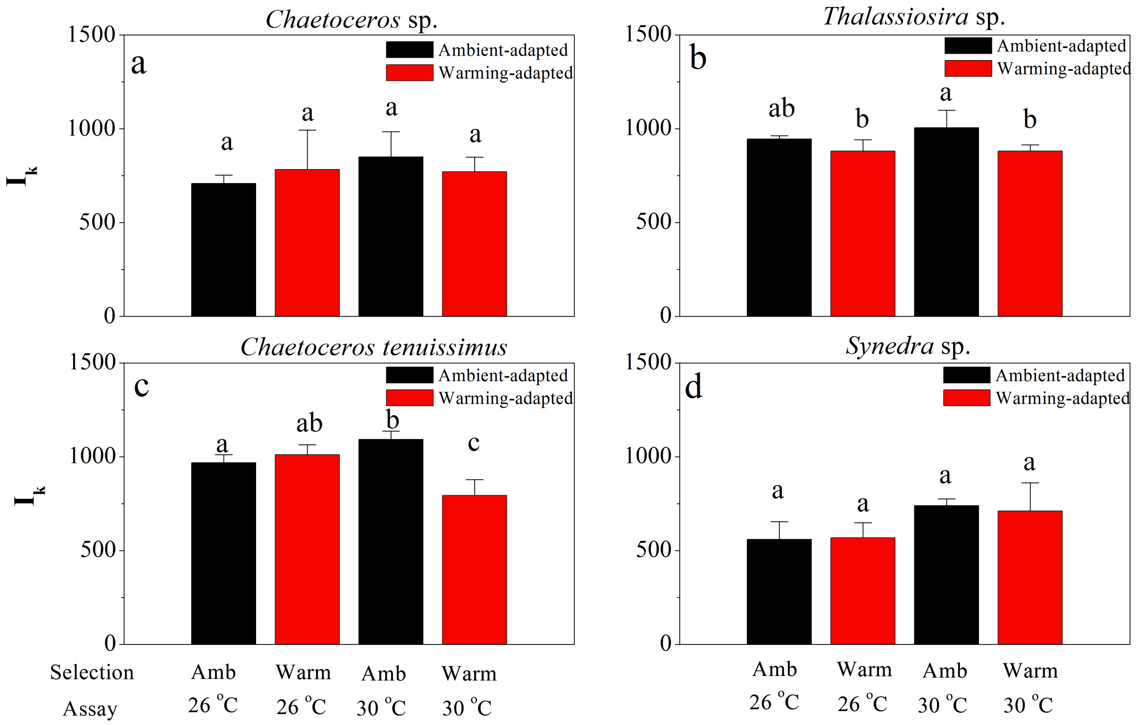

Fig. S6 Photosynthetic responses of saturating light intensity (Ik, μmol photons m^{-2} s^{-1}) of ambient- (26 °C) (black bars) and warming- (30 °C) (red bars) adapted *Chaetoceros* sp. (a), *Thalassiosira* sp. (b), *Chaetoceros tenuissimus* (c) and *Synedra* sp. (d) Cells were exposed to both ambient and warming temperatures. Data are mean ± SE of 4 measurements, and the different letters indicate significant differences between treatments.

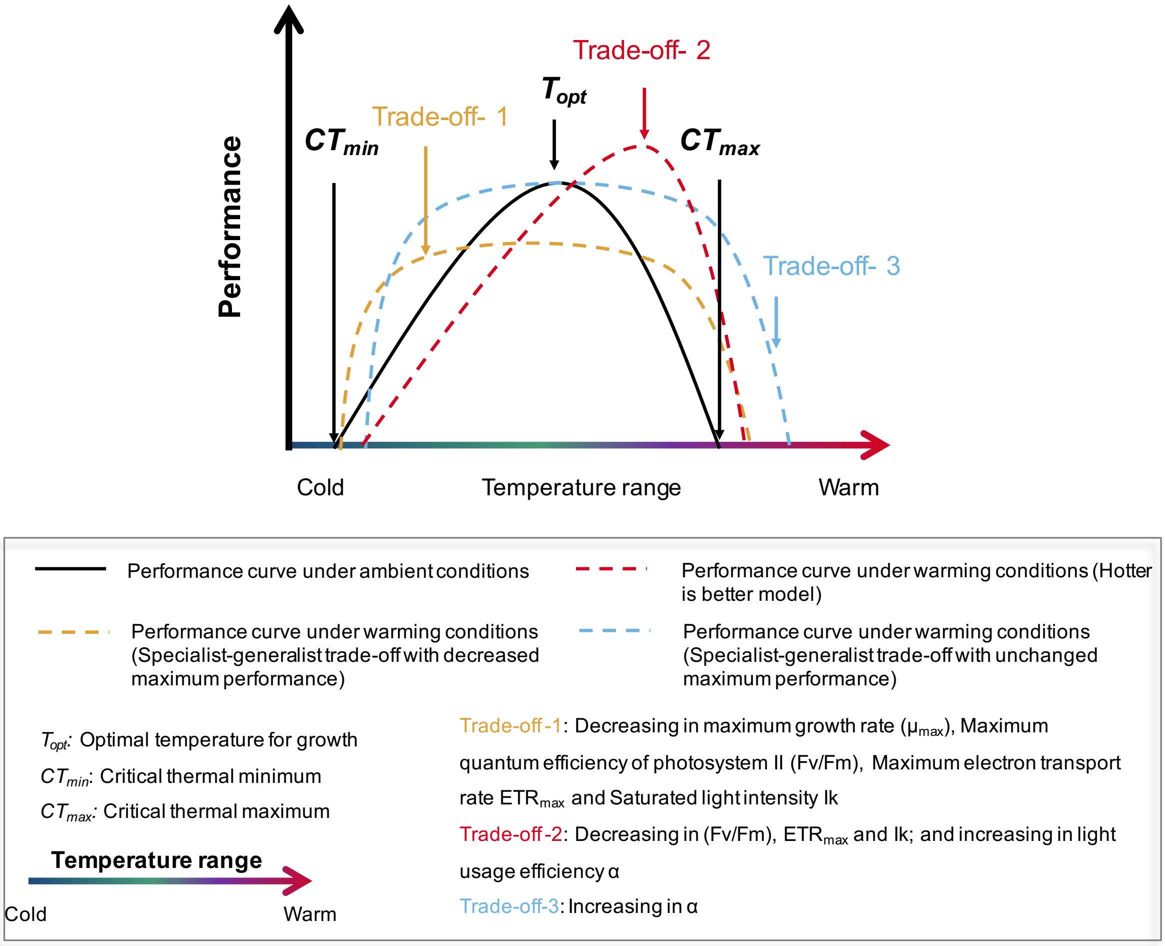

Fig. S7 The graphic summary of the findings in the present study. Two of the species their optimal growth temperature (T_{opt}) and maximum growth rate. The other two diatoms did not increase T_{opt} and growth, but shifted from specialist to generalist increasing their maximum critical thermal limit. All of these thermal adaptations are associated with trade-offs (Trade-off-1,2-3.)

Table. S1 Mixed-effects model analysis of *Chaetoceros* sp., *Thalassiosira* sp., *Chaetoceros tenuissimus* and *Synedra* sp.. DF = Degrees of freedom (numerator, denominator).

Species	Source	numDF	denDF	*F*	*p*
Chaetoceros sp.	Selection day	1	377	16.45	0.0001
	Temperature	1	377	42.61	<0.0001
	Selection day* Temperature	1	377	189.34	<0.0001
Thalassiosira sp.	Selection day	1	377	0.084	0.7722
	Temperature	1	377	142.56	<0.0001
	Selection day* Temperature	1	377	2.554	0.1109
Chaetoceros tenuissimus	Selection day	1	353	1129.9	<0.0001
	Temperature	1	353	543.58	<0.0001
	Selection day* Temperature	1	353	39.00	<0.0001
Synedra sp.	Selection day	1	185	34.996	<0.0001
	Temperature	1	185	29.847	<0.0001
	Selection day* Temperature	1	185	2.924	0.0889

Table. S2 Students' *t*-test of effects of selection temperature on T_{opt} (optimal temperature for growth, °C), μ_{max} (maximum growth rate, d^{-1}), CT_{min} (critical thermal minimum, °C), CT_{max} (critical thermal maximum, °C) and B_{80} (80% performance breadth, °C) in four tested species.

Species	Parameter	t	*F*	*p*
Chaetoceros sp.	T_{opt}	1.65353	6	0.14931
	μ_{max}	-0.18373	6	0.86028
	CT_{min}	-2.84245	6	0.02947
	CT_{max}	-7.66131	6	<0.001
	B_{80}	-2.11773	6	0.07853
Thalassiosira sp.	T_{opt}	-1.15079	6	0.29361
	μ_{max}	2.76314	6	0.03272
	CT_{min}	-2.01456	6	0.09058
	CT_{max}	-7.42894	6	<0.001
	B_{80}	-3.77268	6	0.00926
Chaetoceros tenuissimus	T_{opt}	-12.01129	6	<0.001
	μ_{max}	-12.01917	6	<0.001
	CT_{min}	-6.07463	6	<0.001
	CT_{max}	-4.58271	6	0.00376
	B_{80}	7.54981	6	<0.001
Synedra sp.	T_{opt}	-29.62575	6	<0.001
	μ_{max}	-2.63534	6	0.03878
	CT_{min}	1.03152	6	0.34207
	CT_{max}	0.67014	6	0.52770
	B_{80}	2.39403	6	0.05373

References

1. Eilers, P. H. C. & Peeters, J. C. H. A model for the relationship between light intensity and the rate of photosynthesis in phytoplankton. *Ecol. Model* **42**, 199-215 (1988).
2. National Climatic Data Center. GHRSST Level 4 AVHRR_OI Global Blended Sea Surface Temperature Analysis. 1st ed. Doi:10.5067/GHAAO-4BC01
